# Techniques for RNA extraction from cells cultured in starPEG–heparin hydrogels

**DOI:** 10.1098/rsob.200388

**Published:** 2021-06-02

**Authors:** Anna Jaeschke, Nicholas R. Harvey, Mikhail Tsurkan, Carsten Werner, Lyn R. Griffiths, Larisa M. Haupt, Laura J. Bray

**Affiliations:** ^1^ Institute of Health and Biomedical Innovation, Queensland University of Technology (QUT), Kelvin Grove, Australia; ^2^ Genomics Research Centre, Centre for Genomics and Personalised Health, School of Biomedical Sciences, Queensland University of Technology (QUT), Kelvin Grove, Australia; ^3^ ARC Training Centre for Cell and Tissue Engineering Technologies, Queensland University of Technology (QUT), Kelvin Grove, Australia; ^4^ School of Mechanical, Medical and Process Engineering, Science and Engineering Faculty, Queensland University of Technology (QUT), Brisbane, Australia; ^5^ Leibniz Institute of Polymer Research Dresden, Saxony, Germany

**Keywords:** three-dimensional cell culture, RNA extraction, hydrogels, heparin

## Abstract

Three-dimensional (3D) cell culture models that provide a biologically relevant microenvironment are imperative to investigate cell–cell and cell–matrix interactions *in vitro*. Semi-synthetic star-shaped poly(ethylene glycol) (starPEG)–heparin hydrogels are widely used for 3D cell culture due to their highly tuneable biochemical and biomechanical properties. Changes in gene expression levels are commonly used as a measure of cellular responses. However, the isolation of high-quality RNA presents a challenge as contamination of the RNA with hydrogel residue, such as polymer or glycosaminoglycan fragments, can impact template quality and quantity, limiting effective gene expression analyses. Here, we compare two protocols for the extraction of high-quality RNA from starPEG–heparin hydrogels and assess three subsequent purification techniques. Removal of hydrogel residue by centrifugation was found to be essential for obtaining high-quality RNA in both isolation methods. However, purification of the RNA did not result in further improvements in RNA quality. Furthermore, we show the suitability of the extracted RNA for cDNA synthesis of three endogenous control genes confirmed via quantitative polymerase chain reaction (qPCR). The methods and techniques shown can be tailored for other hydrogel models based on natural or semi-synthetic materials to provide robust templates for all gene expression analyses.

## Graphical abstract

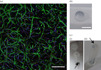

## 1. Introduction

Physiologically relevant *in vitro* models considering the cellular, biochemical and biomechanical aspects of the tissue microenvironment are essential to study the molecular mechanism of diseases such as cancer [1,2]. Hydrogels are commonly used to mimic the extracellular matrix (ECM) in three-dimensional (3D) cell culture techniques [3,4]. Semi-synthetic star-shaped poly(ethylene glycol) (starPEG)–heparin hydrogels have been used for 3D cell culture applications as they are highly tuneable in their biochemical and biomechanical properties. Integrated RGD (arginine–glycine–aspartate) motifs provide cell binding sites, and matrix metalloproteinase (MMP)-responsive sequences allow for cells to locally remodel the microenvironment. Signalling cues, such as growth factors, can be embedded using the negative charge of the heparin molecules [5]. Therefore, starPEG–heparin hydrogels have been used for a variety of research applications such as Alzheimer's disease modelling [6], cartilage regeneration [7] and *in vitro* cancer modelling [8–10]. In particular, these hydrogels are ideal models for tumour angiogenesis studies as their nature allows for vascular cells to extend, interconnect and form networks *in vitro* [11–13].

The biological response of cells to a particular stimulus, such as a treatment, the presence of other cells and cell types, or alterations to microenvironment, can be reflected by changes in gene expression. Such differences can be observed by quantitative polymerase chain reaction (qPCR), northern blotting, high-throughput next-generation RNA sequencing (RNASeq) or whole-transcriptome sequencing. These methods require RNA preparations of high quality. The presence of hydrogel residues (for example, polysaccharides) can impact the use of RNA in these downstream applications [14]. Large polysaccharide fragments can physically capture nucleic acids through electrostatic interactions resulting in reduced or inconsistent RNA yields [15,16]. For starPEG–heparin hydrogels, the presence of heparin in the RNA preparation is of concern. Spin column isolation methods are based on electrostatic interactions between the negatively charged RNA backbone and the silica-membrane of the column. Heparin is also highly negatively charged and can therefore be extracted along with the RNA. Studies have shown that heparin competitively inhibits enzymes such as DNA polymerases [17–19] as well as reverse transcriptases [20,21], presenting a challenge for downstream applications such as qPCR [18,19]. Moreover, significant RNA degradation has been reported following RNA extraction and purification from cells encapsulated in synthetic PEG hydrogels [22].

For RNA isolation, the cell lysis protocol is crucial, as to achieve cell disruption the hydrogel must also be disrupted. In addition, handling of cells can change their expression profile within minutes, requiring disruption of the gels along with cell lysis to occur in parallel to limit any effects on expression profiles. Therefore, the standard approach of digesting gels with collagenase [5] followed by cell lysis was deemed unsuitable for extracting RNA from the embedded cells.

Here, we compared two protocols which combined chemical and mechanical disruption with either flash-freezing or prolonged storage at −80°C. Subsequently, we tested three different RNA purification techniques based on a column, enzyme or a combination approach. We evaluated RNA yield and quality as well as the performance of cDNA synthesized from the various RNA preparations in real-time qPCR (RT-qPCR).

## 2. Material and methods

### 2.1. Cell culture

HUVECs were isolated as previously described [23] and expanded in endothelial cell growth medium-2 (EGM-2) (PromoCell, BanksiaScientificCompany, Bulimba, Queensland, Australia). Cancer-associated fibroblasts (CAFs) were kindly provided by the Prostate Cancer Research Group, Department of Anatomy and Developmental Biology, Monash University [24]. The fibroblasts were cultured in RPMI 1640 media (no phenol red) (Gibco, ThermoFisherScientific, Scoresby, Victoria, Australia) supplemented with 10% fetal bovine serum (FBS) (Gibco, ThermoFisherScientific), 1 nM testosterone (Sigma-Aldrich, CastleHill, New South Wales, Australia), 10 ng ml^−1^ FGF-2 (MiltenyiBiotec, MacquariePark, New South Wales, Australia), 100 U penicillin and 100 µg ml^−1^ streptomycin (Gibco, ThermoFisherScientific). All cells were maintained at 37°C in a humidified incubator containing 5% CO_2_, with media changes every 2–3 days.

### 2.2. Preparation of hydrogel cultures

Three-dimensional co-cultures were obtained using hydrogels comprising synthetic starPEG and maleimide-functionalized heparin, as described previously [5,11]. Briefly, co-cultures of HUVECs and CAFs were seeded into hydrogels at a density of 6 × 10^6^ and 6 × 10^5^ cells ml^−1^ hydrogel, respectively. Vascular endothelial growth factor (VEGF) (Peprotech, Lonza, MountWaverly, Victoria, Australia), human fibroblast growth factor 2 (FGF-2) and stromal cell-derived factor 1 (SDF-1) (MiltenyiBiotec, MacquariePark, New South Wales, Australia) were included into the gel at a concentration of 5 µg ml^−1^ each. Additionally, 2 mol of RGD-SP (H2N-GCWGGRGDSP-CONH2) was added to the gel. A molar ratio of starPEG to heparin-maleimide of 1 : 0.75 was used to obtain a stiffness of approximately 500 Pa (storage modulus). The starPEG–heparin hydrogels were maintained in EGM-2 for 7 days at 37°C in a humidified incubator containing 5% CO_2_.

### 2.3. Preparation of 2D control cultures

HUVECs and CAFs were cultured separately on tissue culture plastic. HUVECs were seeded at 6.8 × 10^3^ cells cm^−2^ and CAFs at 5.5 × 10^3^ cells cm^−2^. The cells were maintained at 37°C in a humidified incubator containing 5% CO_2_, with media changes every 2–3 days for 7 days.

The RNA from both cell types was isolated separately using the Zymo Direct-zol RNA MiniPrep kit (Integrated Sciences, Chatswood, New South Wales, Australia) following the manufacturer's protocol. A mixture of the RNA from the 2D mono-cultures was used representing the cell ratio after 7 days in co-culture (3 : 2, HUVECs : CAFs).

### 2.4. RNA isolation

For the isolation of RNA from cells cultured in starPEG–heparin hydrogels, two different isolation kits were tested which use specific techniques for cell lysis. The *Zymo Direct-zol RNA MiniPrep kit* (Integrated Sciences) includes a lysis step based on TriZOL. When using the *Norgen Total RNA Purification Plus kit* (Millennium Science, Mulgrave, Victoria, Australia), cell lysis was achieved by shock freezing the samples in liquid N_2_, followed by using the lysis buffer present in the kit. Lysates from both preparations were additionally mechanically disrupted by passing the suspension through first a 19-gauge and then a 21-gauge needle. Both RNA isolation kits are based on the principle of using nucleic acid binding columns. All steps in the manufacturers' protocols were followed. DNA removal was performed by an on-column digestion with DNAse I (Zymo kit) or by passing the cell lysate through a DNA binding column prior to RNA isolation (Norgen kit). A detailed step-by-step protocol of the procedures is outlined in table 1.

**Table 1.** Detailed steps of the RNA extraction methods.

step	Zymo Direct-zol RNA MiniPrep kit	Norgen Total RNA Purification Plus kit
1.	Collect hydrogels in a low-binding tube. Briefly dip in PBS to wash.	
2.	Add 100 µl TriZOL reagent per 1 hydrogel.	Add 100 µl RL-buffer reagent (provided in the kit) per 1 hydrogel.
3.	Incubate on ice for 20–30 min.	Flash-freeze in liquid N_2_ and disrupt the frozen hydrogels using a spatula. Repeat the flash-freeze if the sample thaws.
4.	Mechanically disrupt the hydrogels by passing through a 19 Gauge needle attached to a 1 ml syringe. Additionally, use a P1000 pipette to disrupt the hydrogels.	Add another 100 µl RL-buffer reagent per 1 hydrogel.
5.	Store at −80°C until column-based RNA extraction.^a^	Mechanically disrupt the hydrogels by passing through a 19 Gauge needle attached to a 1 ml syringe. Additionally, use a P1000 pipette to disrupt the hydrogels.
6.	Thaw the sample on ice for 30–45 min.	Centrifuge at 10 000*g* for 2 min at 4°C.
7.	Mechanically disrupt the hydrogels by passing through a 21 Gauge needle attached to a 1 ml syringe.	Transfer supernatant into a new low-binding tube. Make note of the sample volume.
8.	Vortex for approximately 20–30 s.	Transfer up to 600 µl of the mixture onto a gDNA removal column. Centrifuge at 13 000*g* for 1 min. Retain the flow through. Repeat for volumes >600 µl.
9.	Centrifuge at 10 000*g* for 2 min at 4°C.	Add 60 µl of 95–100% EtOH to 100 µl of sample (volume noted in Step 7) and mix well.
10.	Transfer supernatant into a new low-binding tube. Make note of the sample volume.	Transfer up to 600 µl of the mixture onto an RNA purification column. Centrifuge at 6500*g* for 1 min. Discard the flow through. Repeat for volumes >600 µl.
11.	Add an equal volume of 95–100% EtOH to the sample and mix well.	Centrifuge for 1 min at 13 000*g* to ensure the entire lysate volume has passed through the column.
12.	Transfer up to 700 µl of the mixture onto a Zymo-Spin IIC column. Centrifuge at 13 000*g* for 1 min. Discard the flow through. Repeat for volumes >700 µl.	Add 400 µl of Wash Solution A to the column. Centrifuge at 13 000*g* for 1 min. Discard the flow through.
13.	Transfer the column into a new collection tube and add 400 µl RNA wash buffer. Centrifuge at 13 000*g* for 1 min. Discard the flow through.	Repeat Step 12
14.	Mix 5 µl DNase I with 75 µl DNA Digestion Buffer and transfer directly onto the column matrix. Incubate at room temperature for 15 min.	Repeat Step 12; 3 washing steps in total.
15.	Add 400 µl Direct-zol RNA PreWash buffer and centrifuge at 13 000*g* for 1 min. Discard the flow though.	Centrifuge at 13 000*g* for 2 min to dry the column membrane.
16.	Repeat Step 15	Elution. Transfer the column into a low-binding tube. Add 50 µl of Elution Solution A and centrifuge at 200*g* for 2 min, followed by 1 min at 13 000*g*. Repeat the centrifugation if necessary. A second elution into a different tube is possible.
17.	Add 700 µl RNA wash buffer to the column. Centrifuge for 2 min at 13 000*g*. Discard the flow through.	Use or store the RNA for downstream applications or purify the RNA (see §2.5).
18.	Centrifuge at 13 000*g* for 1 min to dry the column membrane.	
19.	Elution. Transfer the column into a low-binding tube. Add 50 µl DNase/RNase-free water and centrifuge at 13 000*g* for 2 min, repeat the centrifugation if necessary.	
20.	Use or store the RNA for downstream applications or purify the RNA (see §2.5).	

^a^At least 48 h. The additional freeze/thaw step aids the disruption of the hydrogels.

In the following text, preparations from the Zymo Direct-zol RNA MiniPrep kit are labelled with ‘Zymo kit’ and preparations from the Norgen Total RNA Purification Plus kit are labelled with ‘Norgen kit’.

### 2.5. RNA purification

Following extraction, three purification methods were tested for the RNA preparations. Using the OneStep PCR Inhibitor Removal kit (Integrated Sciences), the RNA was passed through a purification column to remove organic compounds from the RNA preparation. The work was carried out according to the manufacturer's instructions. The use of heparinase digestion to purify RNA has been described previously [25]. Based on this protocol, in the second purification method examined, Heparinase I from *Bacteroides eggerthii* (NE BioLabs, Ipswich, MA, USA) in 1× Heparinase Reaction Buffer was added to the RNA and the reaction incubated at 30°C for 1 h. Additionally, a combination of both procedures was tested. Following Heparinase digestion, the OneStep™ PCR Inhibitor Removal kit (Integrated Sciences) was used to remove residues of the enzyme and reaction buffer from the RNA preparation. A detailed step-by-step protocol is described in table 2. To ensure consistency, all purification methods were tested on the same starting material.

**Table 2.** Detailed steps of the RNA purification protocols.

step	heparinase digestion	OneStep PCR Inhibitor Removal kit	heparinase digestion followed by OneStep PCR Inhibitor Removal kit
1.	Dilute the 10× reaction buffer in RNA. Add DNase/RNase-free water if necessary.	Prepare the Zymo-Spin IV-HRC column by centrifugation at 8000*g* for 3 min.	Dilute the 10× reaction buffer in RNA. Add DNase/RNase-free water if necessary.
2.	Supplement the reaction mix with 1 µl (40 U) RNAseOut (Invitrogen, ThermoFisher).	Place the column in a clean low-binding tube.	Supplement the reaction mix with 1 µl (40U) RNAseOut (Invitrogen, ThermoFisher).
3.	Add 12 U (1 µl) of Heparinase I from *Bacteroides eggerthii* (NE BioLabs) and mix well.	Add 1 µl (40 U) RNAseOut (Invitrogen, ThermoFisher) to the RNA.	Add 12 U (1 µl) of Heparinase I and mix well.
4.	Incubate at 30°C for 1 h.	Add the RNA onto the column and centrifuge at 8000*g* for 1 min.	Incubate at 30°C for 1 h.
5.	Use or store the RNA for downstream applications.	Use or store the RNA for downstream applications.	Prepare the Zymo-Spin IV-HRC column by centrifugation at 8000*g* for 3 min.
6.			Place the column in a clean low-binding tube.
7.			Add 1 µl (40 U) RNAseOut (Invitrogen, ThermoFisher) to the RNA.
8.			Add the RNA onto the column and centrifuge at 8000*g* for 1 min.
9.			Use or store the RNA for downstream applications.

### 2.6. RNA quality

The quality of the RNA was assessed by UV–Vis spectrophotometry (NanoDrop) as well as by chip-based capillary electrophoresis (Bioanalyser).

For the spectrophotometrical measurements, 2 µl of undiluted RNA was pipetted onto a NanoDrop 1000 instrument (ThermoFisherScientific). Measurements were carried out using the NanoDrop 1000 software, v. 3.8.1. Bioanalyser measurements were performed on an Agilent Bioanalyzer 2100 (Agilent Technologies,) using an RNA Pico Kit (Agilent Technologies) according to the manufacturer's protocol. Additionally, qPCR was used to assess the downstream performance of the various RNA preparations. For each RNA sample, 1 μg was reverse transcribed to cDNA using SuperScript IV VILO (Invitrogen, ThermoFisher Scientific) according to the manufacturer's instructions. All incubation steps were carried using a PTC-200 DNA Engine thermal cycler (BioRad, Gladesville, New South Wales, Australia).

The reference genes ribosomal protein L32 (RPL32), TATA-box binding protein (TBP) and Ubiquitin C (UBC) were then used for the qPCR assay. The reaction set up contained 1× PowerUp SYBR Green Master Mix (Invitrogen, ThermoFisherScientific), 200 nM of each, forward and reverse primer and 4 µl of cDNA. The primer sequences are presented in table 3. Triplicates were prepared for each sample and primer pair using a Qiagility pipetting robot (Qiagen, Chadstone Centre, Victoria, Australia). For qPCR, a QuantStudio7 Flex System (ThermoFisherScientific) was used with the following cycling settings: a hold step for 2 min at 50°C and 10 min at 95°C, followed by 30 cycles of denaturing at 95°C for 15 s and annealing/extending for 1  min at 60°C followed by melt curve acquisition. The cycle threshold (Ct) values were derived from the QuantStudio Real-Time PCR software v. 1.3 (ThermoFisherScientific) with automatic threshold settings applied for each target.

**Table 3.** Primer sequences used for qPCR on different RNA preparations.

primer	sequence (5′–3′)	*T_m_* (°C)
RPL32 forward	CCCCTTGTGAAGCCCAAGA	57.6
RPL32 reverse	GACTGGTGCCGGATGAACTT	57.6
TBP forward	TTAACTTCGCTTCCGCTGGC	58.2
TBP reverse	CGCTGGAACTCGTCTCACTA	56.3
UBC forward	GTGGCACAGCTAGTTCCGT	57.6
UBC reverse	CTTCACGAAGATCTGCATTGTCA	55.3

### 2.7. Statistical analysis

For the statistical analysis of Ct values, Student's *t*-test was performed comparing each extraction/purification combination to the 2D control using R v. 3.6 [26]. Statistical significance was assumed with *p* ≤ 0.05.

## 3. Results

**Figure 1.**
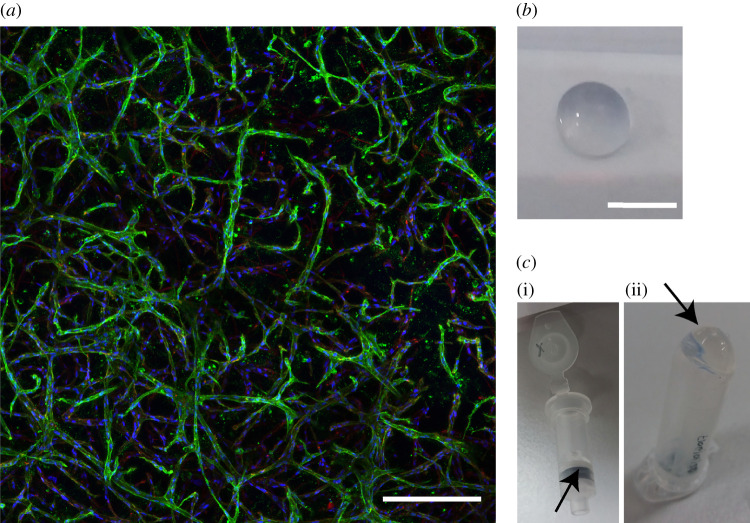
Three-dimensional culture of cells in starPEG–heparin hydrogels. (*a*) Representative maximum intensity projection of a *z*-stack confocal image depicting the cells in a 3D microenvironment. Blue, DAPI; green, CD31; red, F-actin. Scale bar, 250 µm. (*b*) Photograph of a starPEG–heparin hydrogel. Scale bar, 5 mm. (*c*) (i) Hydrogel residue (arrowhead) on an RNA isolation column after centrifugation of the cell lysate. (ii) Observation of a pellet (arrowhead) following centrifugation of the cell lysate.

A representative 2D projection of a *z*-stack confocal image depicting cells cultured within the starPEG–heparin hydrogel is presented in figure 1*a*. Figure 1*b* shows a macroscopic photograph of the hydrogel.

Following the lysis step (table 1), the cell suspension appeared to be completely liquid and a pipette tip did not clog upon pipetting several times. However, upon centrifugation of the lysate through the nucleic acid binding column, transparent hydrogel residuals were observed on the upper surface of the filter (figure 1*c*(i), arrowhead). Upon centrifugation of the lysate at 10 000*g* for 2 min at 4°C, this observation was confirmed by the presence of a pellet, presumably consisting of hydrogel material (figure 1*c*(ii), arrowhead). Small hydrogel pieces present within the sample may clog the column and result in low RNA yield and quality. Therefore, an additional centrifugation step was implemented to remove any residual hydrogel material (Step 9).

**Figure 2.**
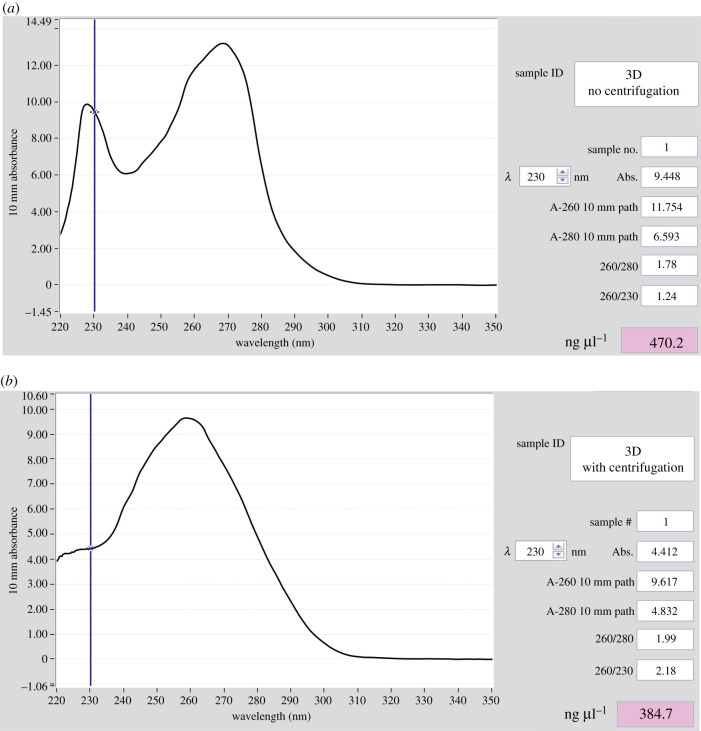
UV–Vis spectrophotometry of RNA extracted with the Zymo kit. (*a*) Without an additional centrifugation step. (*b*) Following an additional centrifugation step prior to loading the cell lysate onto the RNA isolation column.

The results of the UV–Vis spectrophotometry of RNA samples isolated with and without an additional centrifugation step are presented in figure 2. Two distinct peaks, at approximately 230 nm and 260 nm, were visible when no centrifugation step was applied prior to loading the lysate onto the columns (figure 2*a*). Upon the use of the additional centrifugation step, the 230 nm peak was no longer present (figure 2*b*). In addition, the RNA yield was identified to be slightly reduced (approx. 19%) from 470 to 384.7 ng µl^−1^ when the extra centrifugation step was included.

**Figure 3.**
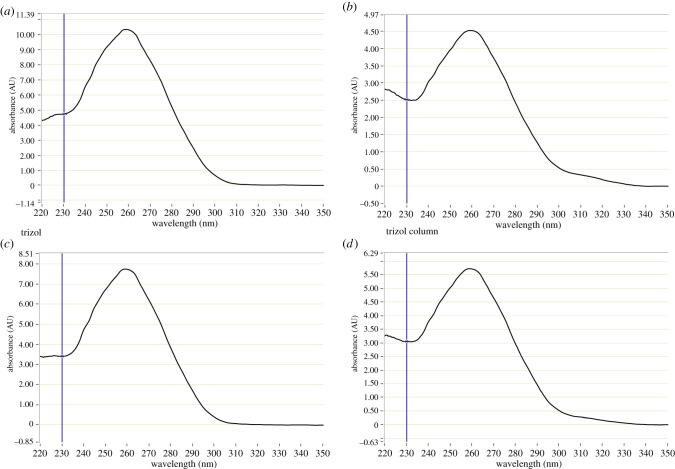
UV–Vis spectrophotometry of RNA isolated with the Zymo kit. (*a*) No further purification. (*b*) Purified with PCR inhibitor removal column. (*c*) Purified using heparinase digestion. (*d*) Purified using heparinase digestion followed by PCR inhibitor removal column.

When the tested purification methods of isolated RNA were examined, a similar general appearance of the UV–Vis spectrophotometry graphs (figure 3; electronic supplementary material, figure S1) was observed for all samples. In particular, a distinct peak at 260 nm was identified in all RNA preparations from the 3D samples resembling that of the 2D control (compare figure 3*a–d* with figure 2*b*).

A closer look at the *A*_260/280_ and *A*_260/230_ ratios for the samples, which are indicators for contamination with proteins or organic compounds, respectively, revealed very little differences between the preparations (table 4). The *A*_260/280_ ratios ranged between 1.88 and 2.04, and the *A*_260/230_ ratios between 1.80 and 2.27. No significant changes were observed between the different isolation kits or the purification protocols.

**Table 4.** *A*_260/280_ and *A*_260/230_ ratios obtained by UV–Vis spectrophotometry (NanoDrop).

protocol	purification method	*A*_260/280_	*A*_260/230_
Zymo kit	none; 2D ctrl	1.96	2.27
Zymo kit	none	2.01	2.19
	heparinase	2.00	1.98
	column	1.88	1.80
	heparinase + column	1.94	1.88
Norgen kit	none	2.10	1.96
	column	1.89	1.80
	heparinase	2.04	2.27
	heparinase + column	1.94	1.81

**Figure 4.**
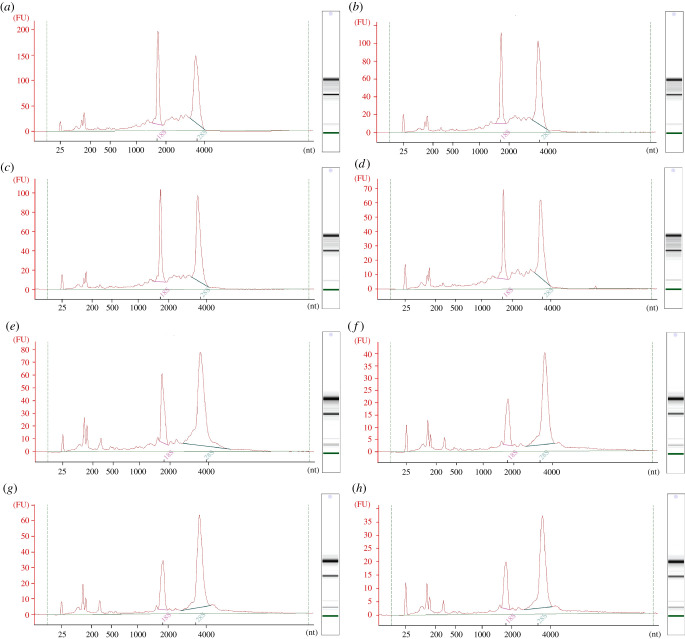
Graphs and gel images obtained from the Bioanalyser. (*a*) Zymo kit, not purified. (*b*) Zymo kit, purified with PCR inhibitor removal column. (*c*) Zymo kit, heparinase digestion. (*d*) Zymo kit, heparinase digestion followed by PCR inhibitor removal column. (*e*) Norgen kit, not purified. (*f*) Norgen kit, purified with PCR inhibitor removal column. (*g*) Norgen kit, heparinase digestion. (*h*) Norgen kit, heparinase digestion followed PCR inhibitor removal column.

A Bioanalyser was used in conjunction with Agilent RNA 6000 PICO chips to further evaluate the quality of the prepared RNA. This identified clear 5S, 18S and 28S peaks as well as clear bands on the virtual electrophoretogram gel in all samples (figure 4). While the 18S and 28S peaks of the RNA isolated with the Zymo kit were of similar heights (figure 4*a*–*d*), in the Norgen kit preparations, a higher 28S peak was observed (figure 4*e*–*h*). This was also reflected by the rRNA ratio which was found to be below 2.0 for the RNA isolated with the Zymo kit but above 2.0, for the samples isolated with the Norgen kit (table 5). Additionally, increased peak sizes below 200 nt indicate a higher abundance of small and miRNA in the RNA preparations isolated with the Norgen kit (figure 4*e*–*h*).

**Table 5.** Bioanalyser data: RNA concentrations, rRNA ratios and RIN. RNA conc. determined using Bioanalyser.

protocol	purification method	RNA conc. (ng µl^−1^)	rRNA ratio 28S/18S	RIN
Zymo kit	none	582.6	1.3	8.2
	heparinase	316.0	1.6	8.5
	column	352.3	1.6	8.4
	heparinase + column	250.9	1.5	7.8
	none	329.5	2.8	8.9
Norgen kit	heparinase	193.8	2.5	9.2
	column	139.8	2.8	8.9
	heparinase + column	125.4	2.7	9.0

RNA integrity number (RIN) is commonly used as an indicator of RNA quality assessment. The algorithm computes a number between 1 and 10 based on various parameters of RNA integrity (ratio between ribosomal peaks), where the higher the number is, the better the RNA quality [27]. The RINs identified for the different RNA preparations ranged between 7.8 and 9.2, which indicates good RNA quality overall. The ratio between 28S and 18S RNA was found to be lower in RNA samples isolated with the TriZol-based method (Zymo kit). These values are summarized in table 5.

As an additional measurement of RNA integrity and quality, qPCR was also used to evaluate the downstream performance of the different RNA preparations. We selected three genes that are commonly used as endogenous controls, ribosomal protein L32 (RPL32), TATA-box binding protein (TBP) and Ubiquitin C (UBC). The cycle threshold (Ct) represents the cycle number at which the fluorescence generated by the reaction crosses a predetermined threshold. Commonly, the Ct value is used as a means to study the relative expression levels of genes of interest between a range of samples. For the current assay, the expression level of the genes examined was not of interest. In this study, changes in Ct values between samples were used as a measure of the presence of inhibitory molecules in the reaction. The Ct values obtained for the 2D control were 23.79 ± 0.25, 21.24 ±0.11 and 14.51 ± 0.06 (mean ± s.d.), for UBC, TBP and RPL32, respectively. For UBC, slightly increased values were found for the samples isolated with the Zymo kit but not the Norgen kit. However, for both TBP and RPL32, the 3D samples showed slightly increased Ct values compared to the 2D controls. The magnitude of the change was dependent on the extraction kit and the purification method. Samples isolated with the Norgen kit showed generally a smaller increase in Ct values (table 6). Overall, there were minor differences in the amplification between the purified RNA samples. No consistent trend in Ct value changes was found for the various purification techniques. In summary, the qPCR analysis exhibited minor differences in downstream performance of the RNA preparations.

**Table 6.** Ct values of UBC, TBP and RPL32. Mean and s.d. (standard deviation) of triplicates, *p*-values derived from Student's *t*-test compared to Zymo kit 2D control.

target	protocol	purification method	mean Ct ± s.d.	% change to 2D ctrl	*p*-value
UBC	Zymo kit	none; 2D ctrl	23.79 ± 0.25	*—*	*—*
	Zymo kit	None	24.51 ± 0.05	+3.03	0.03317*
		heparinase	24.46 ± 0.05	+2.82	0.03645*
		column	24.56 ± 0.05	+3.24	0.02872*
		heparinase + column	24.29 ± 0.19	+2.11	0.05271
	Norgen kit	none	24.19 ± 0.17	+1.68	0.08591
		heparinase	23.91 ± 0.33	+0.50	0.62721
		column	23.74 ± 0.08	−0.21	0.77342
		heparinase + column	23.50 ± 0.13	−1.22	0.17311
TBP	Zymo kit	none; 2D ctrl	21.24 ± 0.11	*—*	*—*
	Zymo kit	none	23.05 ± 0.13	+8.52	0.00120**
		heparinase	23.31 ± 0.20	+9.75	0.00066***
		column	23.12 ± 0.18	+8.85	0.00073***
		heparinase + column	23.00 ± 0.07	+8.29	0.00866**
	Norgen kit	none	22.19 ± 0.09	+4.47	0.01097*
		heparinase	21.82 ± 0.10	+2.73	0.02574*
		column	21.75 ± 0.04	+2.35	0.07481
		heparinase + column	21.81 ± 0.16	+2.68	0.01964*
RPL32	Zymo kit	none; 2D ctrl	14.51 ± 0.06	*—*	*—*
	Zymo kit	none	15.69 ± 0.12	+8.13	0.00081***
		heparinase	15.43 ± 0.10	+6.34	0.00040***
		column	15.74 ± 0.28	+8.48	0.01401*
		heparinase + column	15.29 ± 0.12	+5.38	0.00193**
	Norgen kit	none	15.65 ± 0.06	+7.86	0.00002***
		heparinase	15.40 ± 0.17	+6.13	0.00706**
		column	15.74 ± 0.16	+8.48	0.00236**
		heparinase + column	15.15 ± 0.19	+4.41	0.01968*

*p ≤ 0.05.

**p ≤ 0.01.

***p ≤ 0.001.

## 4. Discussion and conclusion

Bioengineered 3D models are widely used to study cell–cell and cell–matrix interactions within a physiologically relevant microenvironment [2]. The isolation of high-quality RNA is essential to the analysis of gene expression levels in cells cultured in such models. The presence of heparin as a downstream inhibitor of amplification [18–20] is of concern for the extraction of RNA from cells cultured in starPEG–heparin hydrogels. The removal of hydrogel residues as well as the reduction in glycosaminoglycan segments present in the preparation are crucial for obtaining high-quality RNA [18,19,22]. Here, we tested two commercially available kits and combined the disruption of the hydrogels with the cell lysis step on the quality of templates extracted from these hydrogels. Both RNA isolation kits used a combination of chemical and mechanical disruption with an additional freezing step.

We found that the implementation of an additional centrifugation step prior to transferring of the sample to the RNA-binding column significantly increased the quality of RNA obtained. By removing residual hydrogel fragments, contamination of the RNA was reduced and an improvement in RNA quality was observed that surpassed the slight drop in RNA yield observed.

Only minor differences in RNA yield, quality and integrity were identified between the two tested RNA isolation kits. The *A*_260/280_ and *A*_260/230_ ratios obtained by UV-spectrophotometry indicated the absence of contaminants in the RNA preparations from both kits. The rRNA ratios for the isolated RNA samples ranged between 1.3 and 2.8. RNA isolated with the Zymo kit had a lower rRNA ratio overall when compared with the Norgen kit. While preparations with an rRNA ratio of 2.0 are typically considered as high-quality RNA, it has been reported that a low rRNA ratio does not necessarily denote poor mRNA quality [27,28]. The significance of the rRNA ratio may also differ for various downstream applications. For example, for RNAseq, the ratio is likely unimportant as the rRNA is depleted prior to the library preparation. The degradation of the mRNA would be apparent by increased fragmentation detectable through chip-based capillary electrophoresis. This would be visible by more bands or ‘smear’ in the gel images and extra/higher peaks at the lower end of the graph. This was not present for any of the tested samples indicating that all the RNA samples were of good quality. Overall, the minor quality changes which were observed between samples could be due to differences in the RNA concentration of the samples.

Minor differences were observed in the amplification of the three selected endogenous controls used in the study between the 2D control and 3D samples. A slight increase in Ct values was observed in the 3D samples independent of the RNA isolation kit used with the extent being slightly less in the samples isolated with the Zymo kit. However, purification of the RNA did not result in significant changes to the observed Ct values. The slight variations in Ct values are likely due to the starting material rather than the RNA preparations. The samples for the 3D RNA extraction were prepared from the same batch of cells and hydrogels. For the controls, the equivalent type and density of cells were cultured on a 2D tissue culture plastic surface.

The cell density is a crucial factor for RNA yield and the success of subsequent applications. The used cell density resulted in an adequate amount of RNA, between 4 and 20 µg in total. Depending on the application, a significant lower cell number can become a limiting factor for RNA extraction. However, in our hands, the SuperScript IV VILO reverse transcriptase provided successful amplification with efficiency between 90 and 107% with an RNA amount as low as 1 ng (data not shown).

To remove hydrogel residues and heparin contamination, we tested also heparinase digestion, a PCR inhibitor removal column as well as a combination of these steps. No significant improvements in RNA quality were observed with these variations in purification techniques. However, the purification techniques might be of interest for other 3D cell culture techniques. They can be adapted for models with higher matrix density or different glycosaminoglycans, for example, by using other enzymes to purify the RNA.

Both, the Norgen Total RNA Purification Plus kit and the Zymo Direct-zol RNA MiniPrep kit, yielded a good quantity of high-quality RNA suitable for cDNA synthesis and RT-qPCR. Here, we have identified that it is essential to centrifuge the cell–hydrogel suspension prior to RNA isolation, to remove any hydrogel residue. No obvious contamination of the RNA was present after isolation and a subsequent RNA purification step was found to be unnecessary for extracting RNA from cells cultured in starPEG–heparin hydrogels. While the Zymo kit needed a longer handling time due to the additional freezing step, handling of liquid N_2_ with the Norgen kit was found to be of little benefit. By contrast, small RNAs were found to be more abundant in RNA extracted with the Norgen kit which may be of relevance to specific research questions. However, the difference in small RNA composition may be redundant when considering total RNA isolation as there are specific commercial kits for the extraction of small RNA molecules that deliver higher throughput and reproducibility for these specific research questions.

Here, we present two protocols to extract high-quality RNA from glycosaminoglycan-containing hydrogels suitable for gene expression analyses. Both methods and the purification techniques can easily be adapted for other 3D cell culture models based on natural or semi-synthetic materials, for example, hyaluronic acid hydrogels, to produce a robust template for subsequent interrogation.

## Data accessibility

The raw data will be made available upon request.
